# Effects of a Gain-of-Function Mutation in the Voltage-Gated Sodium Channel Gene, and of Dietary α-Linolenic Acid Supplementation, on Whole-Body Metabolism in Drosophila

**DOI:** 10.21203/rs.3.rs-7786194/v1

**Published:** 2025-10-14

**Authors:** Karina Kruth, Junko Kasuya, Victoria Hand, Atulya Iyengar, Toshihiro Kitamoto

**Affiliations:** University of Iowa; University of Iowa; University of Alabama; University of Alabama; University of Iowa

**Keywords:** Epilepsy, Metabolomics, Drosophila, Dietary supplementation, ω-3 Polyunsaturated fatty acid

## Abstract

Epilepsy is a prevalent neurological disorder, and metabolic disturbances are increasingly recognized as key contributors to seizure susceptibility. We profiled whole-body metabolism in the precisely defined, seizure-prone *Drosophila* mutant *para*^*Shu*^, carrying a gain-of-function mutation in the voltage-gated sodium channel gene, and assessed the modulatory impact of dietary α-linolenic acid (ALA). Adult wild-type and mutant females were raised on control or ALA-supplemented diets, and untargeted GC-MS/LC-MS was used to quantify 172 metabolites. The *para*^*Shu*^ mutation led to robust shifts in central carbon metabolism, including increases in glycolytic end products and decreases in TCA and pentose phosphate pathway intermediates. Both outcomes are indicative of mitochondrial dysfunction and reduced NADPH output. Critically, levels of nicotinamide riboside and its derivative nicotinic acid adenine dinucleotide were decreased. This suggests that NAD^+^ biosynthesis was constrained and/or its turnover accelerated. Amino acid networks—particularly those involving tryptophan metabolism—were reorganized in a way that supports NAD^+^ balance and redox regulation, and nucleotide pools were unbalanced. Analysis of fatty-acids revealed high levels of microbially-derived short-chain fatty acids (SCFAs) and medium-chain species, indicative of gut-host interactions. Treatment with ALA partially normalized levels of SCFAs, succinate, 6-phosphogluconate, glycine, and proline, and increased levels of N-methylnicotinamide, consistent with improved redox buffering and dampened signaling by the innate immune pathway. Overall, our data indicate that sodium-channel hyperexcitability elicits coordinated metabolic reprogramming that links mitochondrial dysfunction with redox imbalance and interactions between microbiota and immune pathways, and that dietary ALA lessen these changes. The affected pathways represent testable targets for mechanism-based epilepsy interventions.

## 1. Introduction

Epilepsy is a serious neurological disorder characterized by recurrent, unprovoked seizures caused by abnormal and excessive electrical activity in the brain. According to the 2024 World Health Organization (WHO) Fact Sheets (https://www.who.int/news-room/fact-sheets/detail/epilepsy), approximately 50 million people worldwide are affected by epilepsy, making it one of the most common neurological disorders globally. A variety of factors are known to contribute to the etiology of epilepsy, including brain injuries, tumors, infections, strokes, neurodegenerative diseases, and genetic mutations. However, the mechanisms by which these initial insults transform neuronal circuits into hyperexcitable and unstable networks remain poorly understood. In recent years, metabolomic analyses have been increasingly applied in studies involving both epilepsy patients and rodent models. The goal of these studies was to identify potential diagnostic and prognostic biomarkers, as well as novel therapeutic targets for epilepsy. They also contributed to a better understanding of the mechanisms underlying the genesis and pathophysiology of epilepsy [1–7]. As expected, epilepsy was found to be associated with a range of metabolic alterations [8]. These changes may contribute to seizure generation, arise as a consequence of seizure activity, or both. However, findings from metabolomic studies related to epilepsy are often inconsistent. This is likely due in part to the heterogeneity of epilepsy etiology, and in part to variability in experimental conditions (e.g., differences in genetic backgrounds, physiological states, rearing environments, sampling protocols, and analytical platforms).

The primary objective of this study was to identify changes in metabolism in a well-characterized genetic model of epilepsy, under strictly controlled genetic and environmental conditions. We utilized a powerful model organism, the fruit fly *Drosophila melanogaster*, to determine the metabolic effects of a seizure-inducing mutation in the voltage-gated sodium channel (VGSC) gene [9]. VGSCs are essential for initiating and propagating action potentials, and they do so by responding to membrane depolarization to permit sodium ions to enter neurons [10, 11]. Among the genes that have been implicated in seizure susceptibility, those encoding VGSCs are the most prominently associated with epilepsy disorders [12, 13]. The mammalian genome encodes nine distinct VGSC subtypes [14], and mutations in several of these—particularly *SCN1A*, *SCN2A*, and *SCN8A*—are strongly linked to specific forms of epilepsy. For example, loss-of-function mutations in *SCN1A* cause Dravet syndrome, a severe childhood epilepsy characterized by drug-resistant seizures and sudden unexpected death in epilepsy (SUDEP) [15]. *SCN1A* is predominantly expressed in GABAergic inhibitory interneurons, and its impairment results in disinhibition of excitatory circuits, enhancing neuronal excitability and promoting the synchronized firing that characterizes seizures [16]. Conversely, gain-of-function mutations in *SCN2A* and *SCN8A* lead to excessive or prolonged sodium influx in excitatory neurons, resulting in neuronal hyperexcitability and contributing to early-onset epileptic encephalopathy [17, 18].

Unlike mammals, *Drosophila* has a single VGSC gene, *paralytic* (*para*) [19, 20]. Several features make this organism a valuable platform for dissecting the roles and mechanisms of VGSCs in seizure phenotypes at the molecular, cellular, and organismal levels: the evolutionary conserved structure and function of the VGSC genes; the simplicity of the fly nervous system; and the availability of advanced genetic tools and comprehensive genomic information. *para*^*Shu*^, originally called *Shudderer* [21], is a gain-of-function allele of *para*, in which a missense mutation leads to replacement of a conserved methionine residue in homology domain III of the VGSC protein with isoleucine [9]. Adult *para*^*Shu*^ mutants exhibit a range of dominant seizure-related phenotypes, including characteristic “shuddering” or spontaneous tremors, spontaneous convulsions characterized by synchronized spike discharges, increased susceptibility to electroconvulsive and heat-induced seizures, and ether-induced leg shaking [9]. Additional physical abnormalities include a down-turned wing posture and an indented thorax, both of which are believed to result from excessive muscle contraction caused by neuronal hyperexcitability, given that fly muscles do not produce voltage-gated sodium currents [9].

Both the penetrance and expressivity of neurological phenotypes are highly sensitive to environmental factors because neural circuits are inherently plastic in terms of their development and function. The *para*^*Shu*^ mutant phenotype is no exception. We previous found that dietary supplementation with milk whey significantly reduces the seizure phenotype in *para*^*Shu*^ mutants [22], and that the ω−3 polyunsaturated fatty acid α-linolenic acid (ALA; 18:3n-3) is key to this diet-dependent phenotypic suppression [23]. ALA is present predominantly in plant-based foods such as flaxseed and walnuts [24]. In mammals, it is an essential nutrient and a precursor for long-chain ω−3 fatty acids, including eicosapentaenoic acid (EPA; 20:5n-3) and docosahexaenoic acid (DHA; 22:6n-3) [25]. In *Drosophila*, ALA is not essential [26], but it has been implicated in diverse biological processes, including sensory perception [27] and cholesterol uptake [28]. In spite of these indications that ALA can suppress the *para*^*Shu*^ phenotype, the underlying mechanisms remain unclear.

In this study, we conducted metabolomic analyses using both GC-MS and LC-MS to identify metabolic changes caused by the *para*^*Shu*^ mutation and dietary ALA supplementation. Our discovery that this well-characterized seizure-causing mutation and a defined dietary intervention produce metabolic profiles distinct from their wild-type counterpart provides fundamental insights into the relationship between a genetic predisposition to seizures and metabolism.

## 2. Materials and Methods

### 2.1. Fly stocks and culture conditions

*Drosophila melanogaster* were reared at 25°C and 65% humidity, on a 12-hour light/dark cycle, and on the cornmeal/glucose/yeast/agar medium that was developed by Edward Lewis [29] and modified by Rodney Williamson (Beckman Research Institute of the City of Hope, Duarte, CA). The exact composition of the diet was previously described [22]. The *Canton-S* (*CS*) strain was used as the wild-type control. *para*^*Shu*^[9, 21] was obtained from Mr. Rodney Williamson (Beckman Research Institute of the Hope, CA).

### 2.2. Metabolomic analysis

#### 2.2.1. Sample preparation.

*CS* males were crossed with either virgin *CS* females or virgin heterozygous *para*^*Shu*^ females (*para*^*Shu*^/*FM7*). The resulting progeny were raised on either a standard diet or a diet supplemented with 0.05% (w/v, 1.8 mM) ALA until adulthood. Virgin females (+/+ or *para*^*Shu*^/+) were collected within six hours of eclosion and transferred to vials containing a standard diet, with 10–20 flies per vial. After two days on the standard diet, the flies were collected, flash-frozen in liquid nitrogen, and stored at −80°C for subsequent metabolomic analysis. Whole-body metabolomic analysis was performed using GC-MS and LC-MS at the University of Iowa Metabolomics Core Facility (https://diabetes.medicine.uiowa.edu/research/metabolomics-core-facility).

#### 2.2.2. GC-MS analysis.

Whole-body fly samples were lyophilized for 2 hours prior to homogenization (using bead mill homogenizer) in 18:1 (μL:mg wet tissue weight) ice-cold 2:2:1 methanol/acetonitrile/water extraction buffer containing a mixture of internal standards (D4-citric acid, D4-succinic acid, D8-valine, and U13C-labeled glutamine, glutamic acid, lysine, methionine, serine, and tryptophan; Cambridge Isotope Laboratories). Homogenates were rotated for 1 hour at −20°C. Homogenates were centrifuged for 10 minutes at 21,000 × g, and 150 μL of the cleared metabolite extracts were transferred to autosampler vials and dried using a SpeedVac vacuum concentrator (Thermo). Dried metabolite extracts were reconstituted in 30 μL of 11.4 mg/mL methoxyamine (MOX) in anhydrous pyridine, vortexed for 5 minutes, and heated for 1 hour at 60°C. Next, 20 μL of N,O-Bis(trimethylsilyl)trifluoroacetamide (TMS) was added to each sample, after which samples were vortexed for 1 minute and heated for 30 minutes at 60°C. Derivatized samples were analyzed by GC-MS. For each derivatized sample, 1 μL was injected into a Trace 1300 GC (Thermo) fitted with a TraceGold TG-5SilMS column (Thermo) operating under the following conditions: split ratio = 20:1, split flow = 24 μL/minute, purge flow = 5 mL/minute, carrier mode = Constant Flow, and carrier flow rate = 1.2 mL/minute. The GC oven temperature gradient was as follows: 80°C for 3 minutes, increasing at a rate of 20°C/minute to 280°C, and holding at a temperature at 280°C for 8 minutes. Ion detection was performed using an ISQ 7000 mass spectrometer (Thermo) operated from 3.90 to 21.00 minutes in EI mode (−70eV) using select ion monitoring (SIM).

#### 2.2.3. LC-MS analysis.

Whole-body fly samples were lyophilized and transferred to ceramic bead tubes. For each sample, 18-fold (w/v) extraction solvent (with 9 heavy internal standards) was added and samples were homogenized. After homogenization, samples were rotated at −20°C for 1 hour and then centrifuged at 21,000 × g for 10 minutes. For each sample, 200 μL of the extract supernatant was transferred to microcentrifuge tubes, and the extracts were dried using a SpeedVac Concentrator. Dried extracts were reconstituted in 20 μL acetonitrile/water (1:1 v/v) and vortexed well, and then kept at −20°C overnight. The following day, samples were centrifuged and the supernatant was transferred to LC-MS autosampler vials for analysis. LC-MS data were acquired on a Thermo Q Exactive hybrid quadrupole Orbitrap mass spectrometer with a Vanquish Flex UHPLC system or Vanquish Horizon UHPLC system. The LC column used was a Millipore SeQuant ZIC-pHILIC (2.1 × 150 mm, 5 μm particle size) with a ZIC-pHILIC guard column (20 × 2.1 mm). The injection volume was 2 μL. The mobile phase was as follows: Solvent A (20 mM ammonium carbonate [(NH_4_)_2_CO_3_] and 0.1% ammonium hydroxide (v/v) [NH_4_OH]) and Solvent B (acetonitrile). The flow rate was 0.150 mL/min. The gradient started at 80% B, decreased to 20% B over 20 minutes, returned to 80% B in 0.5 minutes, and was held at 80% for 7 minutes [30]. The mass spectrometer was operated in full-scan, polarity-switching mode from 1 to 20 minutes, with the spray voltage set to 3.0 kV, the heated capillary held at 275°C, and the HESI probe held at 350°C. The sheath gas flow was set to 40 units, the auxiliary gas flow to 15 units, and the sweep gas flow to 1 unit. MS data acquisition was performed across a range of m/z 70–1,000, with the resolution set at 70,000, the AGC target at 1 × 106, and the maximum injection time at 200 ms [30].

#### 2.2.4. Data processing and statistical analysis.

Raw data were analyzed using TraceFinder 5.1 (Thermo). Metabolite identification and annotation required at least two ions (target + confirming), a unique retention time specific to the ions, and the retention time of a reference standard previously determined in-house. A pooled-sample generated prior to derivatization was analyzed at the beginning of the analytical run, at a set interval during the analytical run, and at the end the analytical run, to correct peak intensities using the NOREVA tool [31]. NOREVA-corrected data were then normalized to the sum total of signal per sample to control for extraction, derivatization, and/or loading effects. For short-chain fatty acids (SCFAs), acquired LC-MS data were processed using the Thermo Scientific TraceFinder 4.1 software, and metabolites were identified using the University of Iowa Metabolomics Core facility standard-confirmed, in-house library. Analyte signal was corrected by normalizing to the deuterated analyte signal, and the signal obtained from a processing blank (PB) was subtracted. The processed data were evaluated using functional modules for statistical analysis in MetaboAnalyst 5.0 [32], as well as GraphPad Prism 10 (GraphPad Software, Inc., Boston, MA).

## 3. Results

### 3.1. Alterations in overall whole-body metabolism induced by *para*^*Shu*^ and dietary supplementation with ALA

**Figure 1:**
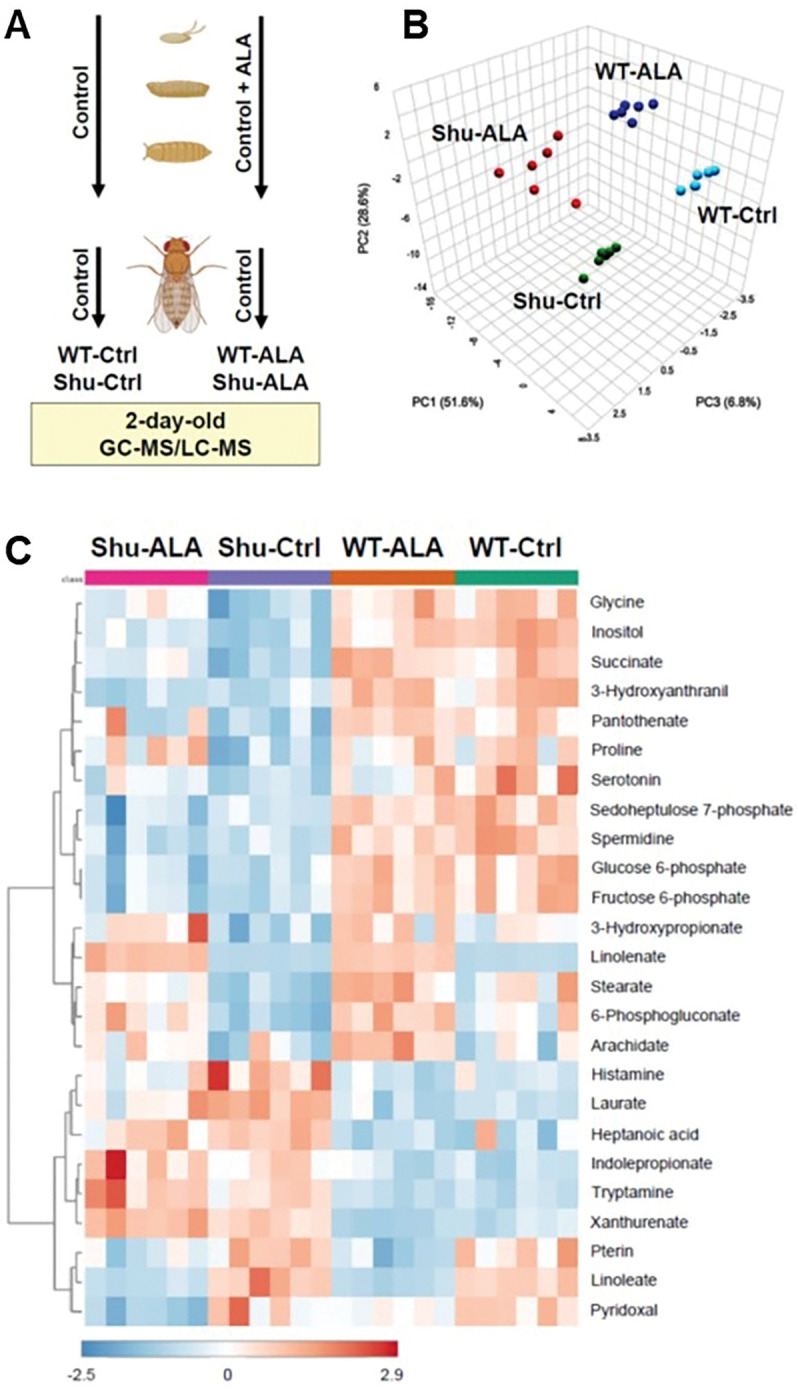
Whole-body metabolomic analysis of control and *para*^*Shu*^ mutant flies with and without ALA treatment. **(A)** Schematic of the experimental design. WT-Ctrl and Shu-Ctrl flies were maintained on a control diet throughout the experiment. WT-ALA and Shu-ALA flies were reared on the ALA diet until eclosion, then transferred to a control diet for two days before analysis. Each group included six biological replicates, and each replicate included approximately 50 flies. **(B)** Three-dimensional principal component scatterplot. **(C)** Heatmap of hierarchical clustering based on the most significantly altered metabolites.

To identify metabolic changes resulting from the *para*^*Shu*^ mutation and dietary ALA supplementation, we performed whole-body metabolomic analysis on adult female *Drosophila* using GC-MS and LC-MS (Fig. 1A). Four experimental groups were analyzed: (1) wild-type *Canton-S* (*CS*) flies on a control diet (WT-Ctrl), (2) *para*^*Shu*^ heterozygotes on a control diet (Shu-Ctrl), (3) *CS* flies on a diet supplemented with ALA (WT-ALA), and (4) *para*^*Shu*^ heterozygotes on a diet supplemented with ALA (Shu-ALA). A total of 172 metabolites were analyzed (Supplementary Table 1). Principal component analysis (PCA) showed that for each group the six biological replicates were tightly clustered and that the four groups were clearly separated (Fig. 1B). Thus, both the *para*^*Shu*^ mutation and ALA supplementation induce reproducible changes in whole-body metabolite profiles and these changes are distinct.

One-way ANOVA was used to compare metabolite levels across the four experimental groups (Supplementary Table 2). Heatmaps of the 25 most significantly altered metabolites highlight the primary effects of the *para*^*Shu*^ mutation and ALA treatment on the metabolome (Fig. 1C): seven of the affected metabolites are directly involved in fatty acid metabolism (linolenate, linoleate, laurate, stearate, heptanoic acid, arachidate, and 3-hydroxypropionate); five are associated with tryptophan metabolism (tryptamine, xanthurenate, 3-hydroxyanthranilic acid, serotonin, and indolepropionate); and four are involved in glycolysis or the pentose phosphate pathway (PPP; fructose 6-phosphate, glucose 6-phosphate, 6-phosphogluconate, and sedoheptulose 7-phosphate).

Given that the primary goal of this study was to identify metabolic changes associated with the hyperexcitability phenotype caused by the *para*^*Shu*^ mutation, as well as with the suppression of *para*^*Shu*^ phenotypes by dietary ALA, we performed two pairwise comparisons: (1) Shu-Ctrl vs. WT-Ctrl and (2) Shu-ALA vs. Shu-Ctrl. These analyses were conducted using Student’s t-tests with a false discovery rate (FDR) threshold of < 0.05. The number of significantly altered metabolites was 55 for Shu-Ctrl vs. WT-Ctrl, and 11 for Shu-ALA vs. Shu-Ctrl (Table 1). Seven metabolites were common to both comparisons (indicated in bold in Table 1). Notably, the levels of all these metabolites changed in opposite directions in response to the *para*^*Shu*^ mutation and ALA treatment, consistent with ALA having suppressive effects on the physiological and behavioral phenotypes of *para*^*Shu*^.

**Figure 2:**
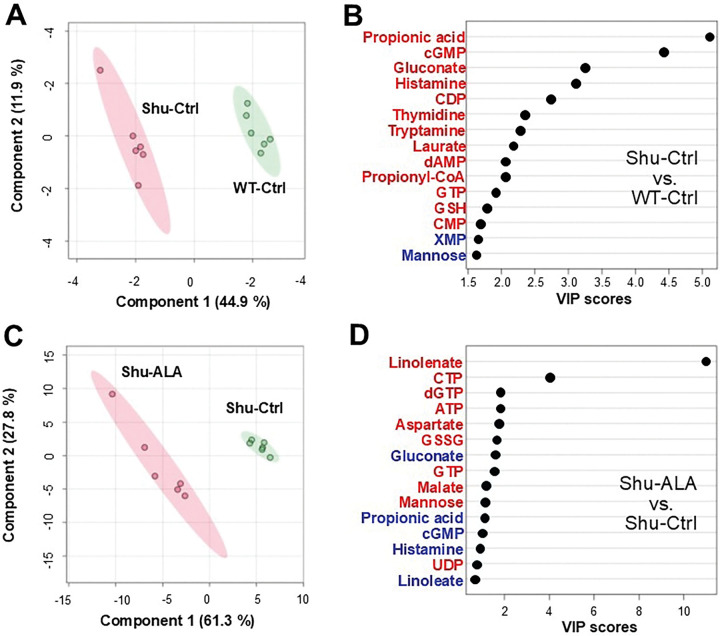
Multivariate analysis of metabolic effects of the *para*^*Shu*^ mutation and dietary ALA supplementation. **(A, C)** Partial least squares discriminant analysis (PLS-DA) score plots comparing Shu-Ctrl and WT-Ctrl flies **(A),** and Shu-ALA and Shu-Ctrl flies **(C). (B, D)** Variable importance in projection (VIP) plots showing the metabolites that contributed most to the group separation observed in **(A)** and **(C)**. Metabolites in red were elevated and those in blue were reduced in Shu-Ctrl compared with WT-Ctrl **(B)**, and in Shu-ALA compared with Shu-Ctrl **(D)**.

Group separation was visualized using Partial Least Squares Discriminant Analysis (PLS-DA; Fig. 2A, C), and the metabolites most responsible for this distinction were identified based on Variable Importance in Projection (VIP) scores (Fig. 2B, D). A VIP score greater than 1.0 is generally considered important for group discrimination. In the comparison between Shu-Ctrl and WT-Ctrl, propionic acid, cGMP, gluconate, and histamine had high VIP scores (5.1, 4.4, 3.2, and 3.1, respectively) indicative of strong contributions to group separation (Fig. 2C). In the comparison between Shu-Ctrl and Shu-ALA, these metabolites likewise had high VIP scores (1.1, 0.99, 1.53, and 0.94, respectively; Fig. 2D), and thus also appear to contribute to separation. Notably, *para*^*Shu*^ and ALA had opposite effects on these metabolites. In the *para*^*Shu*^ mutant the metabolite levels increased whereas in the context of dietary ALA supplementation they decreased (Fig. 2C, D).

**Figure 3:**
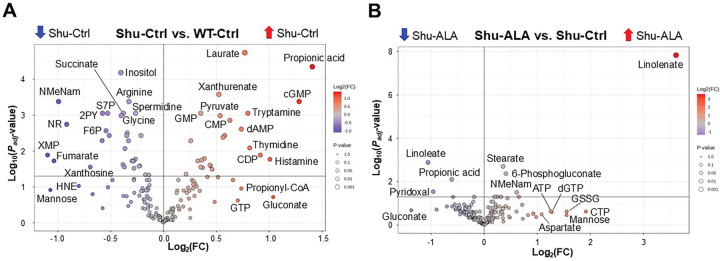
Volcano plots of metabolite changes induced by the *para*^*Shu*^ mutation and ALA supplementation. **(A)** Shu-Ctrl vs. WT-Ctrl flies. **(B)** Shu-ALA vs. Shu-Ctrl flies. Adjusted *P*-value (*P*_*adj*_) and fold change (FC) are indicated. Abbreviations of the labeled metabolites: NMeNam, N-methylnicotinamide; S7P, sedoheptulose 7-phosphate; 2PY, N-methyl-2-pyridone-5-carboxamide; NR, nicotinamide riboside; HNE, 4-hydroxy-2-nonenal; XMP, xanthosine monophosphate; F6P, fructose-6-phosphate; GSSG, glutathione disulfide.

Metabolites that are significantly up- or downregulated by the *para*^*Shu*^ mutation (Fig. 3A) or dietary ALA supplementation (Fig. 3B) were shown using volcano plots. Major metabolic pathways affected by these conditions were identified by pathway analysis using the MetaboAnalyst tool [32]. Applying cutoff criteria of FDR < 0.01 and a pathway impact score (PIS) > 0.3, we identified 18 significantly affected metabolic pathways in the Shu-Ctrl vs. WT-Ctrl comparison (Table 2). Among these, the six pathways with the highest PIS values were: (1) Alanine, aspartate, and glutamate metabolism (PIS = 0.804, FDR = 0.000106); (2) Nicotinate and nicotinamide metabolism (PIS = 0.773, FDR = 0.000593); (3) Pentose phosphate pathway (PPP) (PIS = 0.722, FDR = 8.98 × 10^−5^); (4) Glycine, serine, and threonine metabolism (PIS = 0.667, FDR = 0.00078); (5) Arginine biosynthesis (PIS = 0.629, FDR = 0.00326); and (6) Tryptophan metabolism (PIS = 0.549, FDR = 0.000459). In contrast, no pathways met these stringent criteria in the Shu-ALA vs. Shu-Ctrl comparison. However, when more relaxed cutoffs were applied (FDR < 0.05 and PIS > 0.3), Vitamin B6 metabolism (FDR = 0.026, PIS = 0.33) was found to be significantly affected by ALA in this comparison. In the following sections, we highlight key effects on metabolites within the pathways that are most significantly influenced by the *para*^*Shu*^ mutation or dietary ALA treatment.

### 3.2. Central carbon metabolism

**Figure 4:**
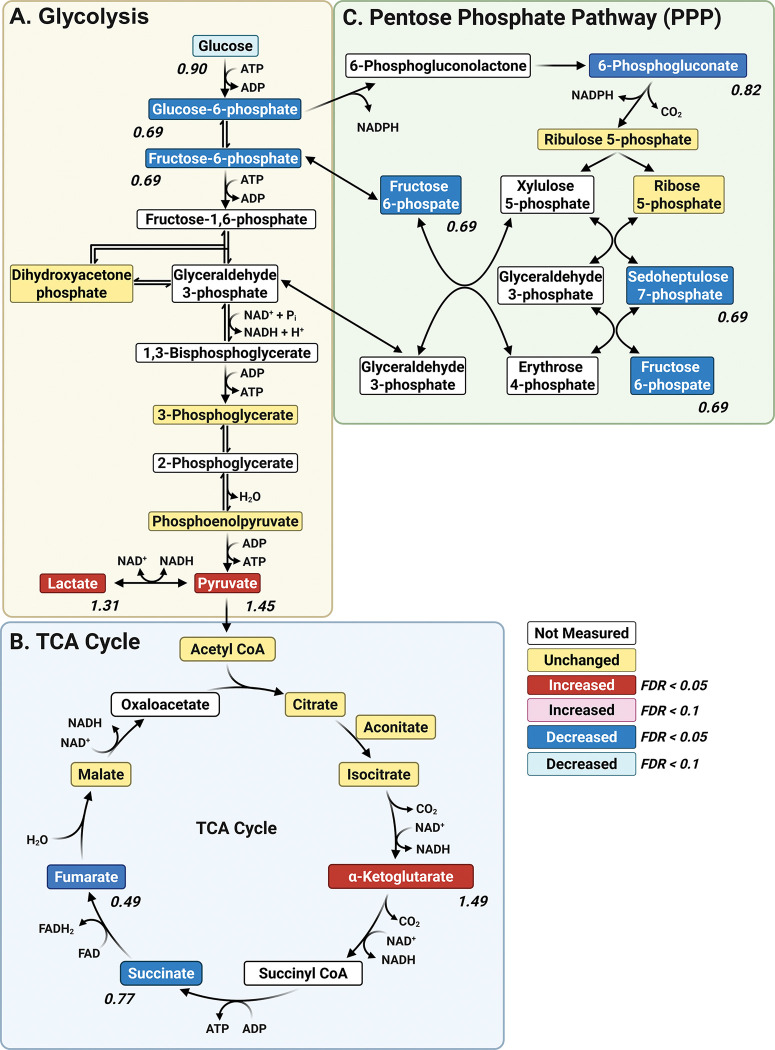
Effects of the *para*^*Shu*^ mutation on central carbon metabolism. Shown are metabolites of central carbon metabolism, including those involved in glycolysis **(A)**, the tricarboxylic acid (TCA) cycle **(B)**, and the pentose phosphate pathway **(C)**. Differences in metabolites in Shu-Ctrl relative to WT-Ctrl flies are indicated as follows: red boxes, upregulated (FDR < 0.05); pink boxes, upregulated (FDR < 0.1); blue boxes, downregulated (FDR < 0.05); cyan boxes, downregulated (FDR < 0.1); yellow boxes, unchanged; white boxes, not measured. Fold change (FC) values are shown, where FC > 1 denotes an increase and FC < 1 denotes a decrease in metabolite levels in Shu-Ctrl flies relative to WT-Ctrl flies.

Central carbon metabolism (CCM, Fig. 4) is a network of biochemical pathways that processes carbon sources to generate energy, biosynthetic precursors, and reducing equivalents. The major CCM pathways include glycolysis, the tricarboxylic acid (TCA) cycle, and the pentose phosphate pathway (PPP)[33], all of which were significantly affected in *para*^*Shu*^ mutants (Table 2). The primary end product of glycolysis is pyruvate, and under anaerobic conditions it is converted to lactate through fermentation; this reaction regenerates NAD^+^ to allow glycolysis to continue (Fig. 4A). Levels of both pyruvate (FC = 1.44, FDR = 0.001) and lactate (FC = 1.30, FDR = 0.016) were significantly higher in *para*^*Shu*^ mutants than wild-type flies, indicating that glycolytic flux is high in the mutants. However, levels of upstream intermediates of glycolysis, such as glucose 6-phosphate (FC = 0.66, FDR = 0.0051) and fructose 6-phosphate (FC = 0.68, FDR = 0.0027), were lower. This could potentially be due to increased consumption downstream, which would lead to substrate depletion. It is also possible that alternative sources of pyruvate are utilized, for example the three-carbon amino acids alanine, serine, and cysteine. Within the TCA cycle (Fig. 4B), levels of succinate (FC = 0.76, FDR = 0.00088) and fumarate (FC = 0.48, FDR = 0.018) were lower in the *para*^*Shu*^ mutants, suggesting that the pathway is disrupted at the middle or late stage. Conversely, α-ketoglutarate was elevated in the mutant (FC = 1.49, FDR = 0.0037); this could reflect either a reduction in the conversion of α-ketoglutarate to downstream TCA intermediates or an increase in glutaminolysis (with glutamine metabolized to glutamate and subsequently to α-ketoglutarate). Collectively, these changes suggest that flux of the TCA cycle is impaired. The *para*^*Shu*^ mutants also had significantly lower levels of 6-phosphogluconate (FC = 0.82, FDR = 0.018) and sedoheptulose 7-phosphate (FC = 0.69, FDR = 0.00088), both of which are PPP intermediates, as well as fructose 6-phosphate (FC = 0.68, FDR = 0.0027), a metabolite that is both a glycolytic intermediate and a product of the non-oxidative branch of the PPP (Fig. 4C).

Treatment of *para*^*Shu*^ mutants with ALA partially reversed some of the effects of *para*^*Shu*^ on the CCM. Dietary ALA supplementation led to increased levels of succinate (FC = 1.14, FDR = 0.049) and 6-phosphogluconate (FC = 1.32, FDR = 0.0042), suggesting that function of the TCA cycle and PPP were improved. However, it did not lead to significant increases in levels of core glycolytic intermediates (FDR < 0.05), suggesting that that ALA does not substantially affect glycolysis.

### 3.3. Amino acid metabolism

The *para*^*Shu*^ mutation profoundly alters amino acid metabolism. Of the 55 metabolites that differed significantly (FDR < 0.05) between *para*^*Shu*^ and wild-type flies, 18 are directly involved in amino acid metabolism (marked with an asterisk in Table 1A). Among them, those that were present at significantly higher levels in *para*^*Shu*^ flies were the neurotransmitter-related metabolites histamine (FC = 2.0, FDR = 0.017) and gamma-aminobutyrate (GABA; FC = 1.25, FDR = 0.019). Another was the methionine derivative homocysteine (FC = 1.35, FDR = 0.025). Another set of amino acids and their derivatives were lower in *para*^*Shu*^ mutants. These included alanine (FC = 0.90, FDR = 0.046), beta-alanine (FC = 0.83, FDR = 0.016), glutamine (FC = 0.88, FDR = 0.045), glycine (FC = 0.75, FDR = 0.001), proline (FC = 0.81, FDR = 0.019), arginine (FC = 0.79, FDR = 0.0004), and cadaverine, a lysine derivative (FC = 0.85, FDR = 0.026).

**Figure 5:**
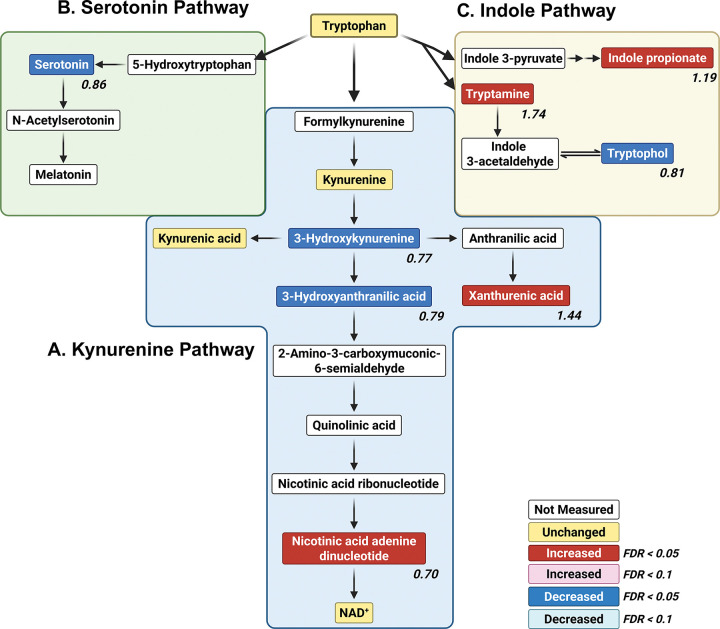
Effects of the *para*^*Shu*^ mutation on metabolites in the tryptophan pathway. Tryptophan metabolites in the kynurenine (A), serotonin (B), and indole (C) pathways are shown. Differences between Shu-Ctrl and WT-Ctrl flies are indicated as follows: red boxes, upregulated (FDR < 0.05); pink boxes, upregulated (FDR < 0.1); blue boxes, downregulated (FDR < 0.05); cyan boxes, downregulated (FDR < 0.1); yellow boxes, unchanged; white boxes, not measured. Fold change (FC) values are shown, where FC > 1 denotes an increase and FC < 1 denotes a decrease in metabolite levels in Shu-Ctrl flies relative to WT-Ctrl flies.

Beyond playing a role in protein synthesis, tryptophan is a precursor to several important bioactive compounds, including NAD^+^, which supports energy metabolism and redox homeostasis [34]) and serotonin, which is both a neurotransmitter and a neuromodulator. Tryptophan is metabolized via three major pathways—the kynurenine, serotonin, and indole pathways [35–37]. All of these are severely affected by the *para*^*Shu*^ mutation (Fig. 5). In the kynurenine pathway (Fig. 5A), xanthurenate (FC = 1.44, FDR = 0.00026) levels were higher, whereas those of 3-hydroxykynurenine (FC = 0.76, FDR = 0.023) and 3-hydroxyanthranilic acid (FC = 0.78, FDR = 0.0037) were lower. This suggests that the metabolic processes driving NAD^+^ synthesis are impaired in mutants, potentially disrupting redox regulation. In the serotonin pathway (Fig. 5B), serotonin levels were lower (FC = 0.86, FDR = 0.036), suggesting that serotonergic signaling was diminished. In the indole pathway (Fig. 5C), levels of tryptamine and indolepropionate were higher (tryptamine: FC = 1.74, FDR = 0.0088; indolepropionate: FC = 1.19, FDR = 0.0051), whereas levels of tryptophol were lower (FC = 0.81, FDR = 0.019).

The effects of the *para*^*Shu*^ mutation on amino acids were partially reduced by ALA supplementation. Notably, although levels of proline and glycine—which are central to redox balance and neurotransmission—were lower in *para*^*Shu*^ mutants, they were normalized in the context of ALA supplementation (proline: FC = 1.24, FDR = 0.049; glycine: FC = 1.18, FDR = 0.049), showing that dietary ALA helps restore neurochemical and metabolic homeostasis.

### 3.4. Nucleotide metabolism

**Figure 6:**
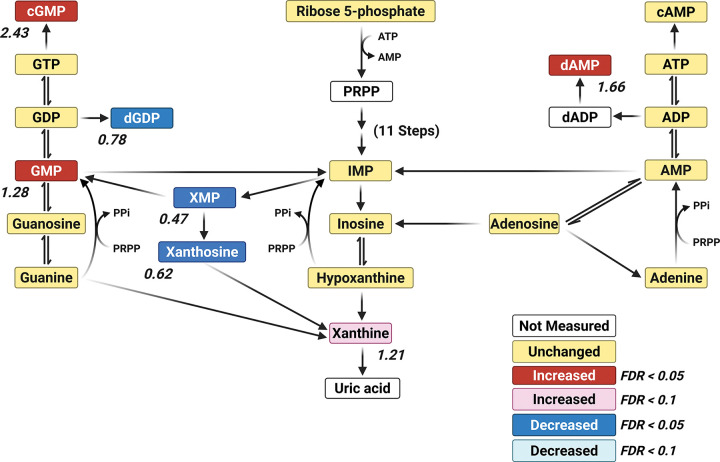
Effects of the *para*^*Shu*^ mutation on metabolites in the purine pathway. Purine metabolites are shown. Differences between Shu-Ctrl and WT-Ctrl flies are indicated as follows: red boxes, upregulated (FDR < 0.05); pink boxes, upregulated (FDR < 0.1); blue boxes, downregulated (FDR < 0.05); cyan boxes, downregulated (FDR < 0.1); yellow boxes, unchanged; white boxes, not measured. Fold change (FC) values are shown, where FC > 1 denotes an increase and FC < 1 denotes a decrease in metabolite levels in Shu-Ctrl flies relative to WT-Ctrl flies. red boxes, upregulated (FDR < 0.05); pink boxes, upregulated (FDR < 0.1); blue boxes, downregulated (FDR < 0.05); cyan boxes, downregulated (FDR < 0.1); yellow boxes, unchanged; white boxes, not measured. Fold change (FC) values are shown, where FC > 1 denotes an increase and FC < 1 denotes a decrease in metabolite levels in Shu-Ctrl flies relative to WT-Ctrl flies.

The metabolomic profile of the *para*^*Shu*^ mutant also revealed widespread perturbations in nucleotide metabolism, including both the purine and pyrimidine pathways. Levels of several pyrimidine nucleotides were higher. This was the case for CMP (FC = 1.54, FDR = 0.00139), UMP (FC = 1.48, FDR = 0.00413), CDP (FC = 1.89, FDR = 0.0129), and dCMP (FC = 1.16, FDR = 0.0439). Similar trends were observed for the nucleoside thymidine (FC = 1.76, FDR = 0.00815) and its precursor thymine (FC = 1.29, FDR = 0.029). These findings suggest that the biosynthesis and turnover of pyrimidines were increased overall. In contrast to the observed trend for an increase in pyrimidine levels, the effects on purine metabolism were inconsistent (Fig. 6). Levels of both GMP (FC = 1.28, FDR = 0.000884) and its signaling derivative cGMP (FC = 2.4317, FDR = 0.00041612) were markedly increased, consistent with a shift toward guanine nucleotide accumulation and increased cGMP-mediated signaling. However, levels of several purine intermediates were significantly lower. These included XMP (FC = 0.47, FDR = 0.0129), xanthosine (FC = 0.62, FDR = 0.0278), and dGDP (FC = 0.776). Notably, levels of cAMP were higher in the mutant (FC = 1.66, FDR = 0.00244). The contrast between this observation and the decrease in dGDP levels highlights that the deoxynucleotide balance is disrupted.

### 3.5. Fatty acid metabolism

**Figure 7:**
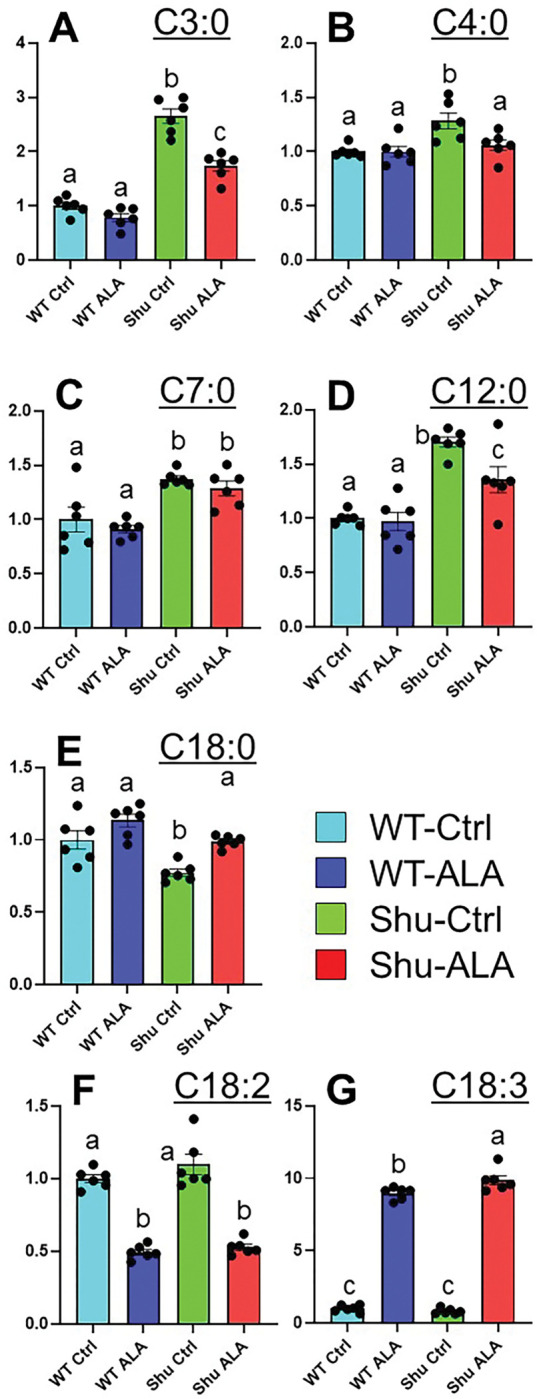
Effects of the *para*^*Shu*^ mutation and dietary ALA supplementation on levels of fatty acids. Relative levels of fatty acids in WT-Ctrl (cyan), WT-ALA (navy), Shu-Ctrl (green), and Shu-ALA (red) flies, shown as mean ± SEM for six biological replicates. Statistical differences are indicated by lowercase letters. Abbreviations: C3:0, propionic acid; C4:0, butyric acid; C7:0, heptanoic acid; C12:0, lauric acid; C18:0, stearic acid; C18:2, linoleic acid; C18:3, α-linolenic acid.

The *para*^*Shu*^ mutation and ALA supplementation also noticeably influenced fatty-acid metabolism. Among the saturated fatty acids, five were markedly affected. Specifically, levels of two SCFAs, propionic acid (C3:0) (FC = 2.65, FDR = 4.46 × 10^− 5^) and butyric acid (C4:0) (FC = 1.28, FDR = 0.0179), and two medium-chain fatty acids (MCFAs), heptanoic acid (C7:0) (FC = 1.37, FDR = 0.0323) and lauric acid (C12:0) (FC = 1.31, FDR = 0.0162), were markedly higher in *para*^*Shu*^ mutants (Fig. 7A–D). In contrast, levels of the long-chain fatty acid (LCFA) stearic acid (C18:0) (FC = 0.773, FDR = 0.0278) were lower (Fig. 7E). ALA treatment partially reversed most of these effects in *para*^*Shu*^ mutants. Levels of propionic acid and butyric acid were 35% and 11% lower, respectively, in ALA-treated *para*^*Shu*^ mutants (Fig. 7A, B). Levels of lauric acid and stearic acid in ALA-treated *para*^*Shu*^ mutants were also approaching to those in wild-type flies (Fig. 7D, E). The exception was heptanoic acid, which remained elevated in *para*^*Shu*^ mutants compared with wild-type flies, regardless of diet type (Fig. 7C).

We also examined effects on linolenate and linoleate, the ionic forms of ω−3 ALA and ω−6 linoleic acid (LA), respectively. As expected, linolenate levels were significantly higher after dietary ALA supplementation in both wild-type flies (FC = 8.96, FDR = 3.67 × 10^−10^) and *para*^*Shu*^ mutants (FC = 12.2, FDR = 1.48 × 10^− 8^ (Fig. 7G). Notably, although linolenate and linoleate cannot be interconverted enzymatically or chemically in the body, linoleate levels were approximately 50% lower in both wild-type flies and the mutants fed the ALA-supplemented diet (Fig. 7F). These findings suggest that the ω−3 and ω−6 fatty acid metabolic pathways strongly regulate one another.

## 4. Discussion

### 4.1. Broad metabolic reprogramming in *para*^*Shu*^ mutants: alterations in energy metabolism and redox homeostasis

Our whole-body metabolomic profiling revealed that *para*^*Shu*^ mutants undergo broad metabolic reprogramming that leads to significant alterations in both energy metabolism and redox regulation. Lower levels of succinate and fumarate in the mutants suggest that the TCA cycle was disrupted at the succinate–fumarate-malate branch (Fig. 4B). Notably, the 51% reduction in fumarate levels indicates that the activity of succinate dehydrogenase (SDH), which catalyzes the oxidation of succinate to fumarate, was severely impaired. SDH is unique in that it directly participates in both the TCA cycle and the mitochondrial electron transport chain. Also known as Complex II, SDH transfers electrons to ubiquinone. Consequently, reduced SDH activity limits electron transfer to the respiratory chain, compromises mitochondrial energy production, and may shift cellular metabolism toward increased reliance on glycolysis. In support of this notion, the accumulation of pyruvate and lactate in *para*^*Shu*^ mutants reflects a metabolic shift toward glycolysis (Fig. 4A), reminiscent of the Warburg effect commonly observed in cells under mitochondrial stress or heightened energy demand [38]. Consistent with our findings, enhanced glycolytic activity (evidenced by increased lactate levels) has been reported in both patients with epilepsy and rodent models of epilepsy [8].

We also observed that several PPP intermediates were significantly reduced in *para*^*Shu*^ mutants. These include fructose 6-phosphate, 6-phosphogluconate, and sedoheptulose 7-phosphate (Fig. 4C). These differences suggest that PPP activity decreased, which would reduce the production of NADPH—a cofactor essential for maintaining redox homeostasis. Levels of other metabolites involved in redox regulation and the oxidative stress response were also altered. Notably, levels of the NAD^+^ precursor nicotinamide riboside (FC = 0.529, FDR = 0.00179) and the NAD^+^ nicotinic acid adenine dinucleotide (FC = 0.70, FDR = 0.00369)—a critical redox cofactor—were significantly reduced. This depletion suggests that either NAD^+^ turnover was increased or NAD^+^ biosynthesis was impaired; either could compromise the redox balance and cellular energy status. The accumulation of homocysteine, a sulfur-containing amino acid that promotes oxidative stress (FC = 1.358, FDR = 0.025338), provides further evidence of redox dysregulation in *para*^*Shu*^ mutants.

The *para*^*Shu*^ mutation also caused significant reductions in levels of several amino acids (Table 1) that are essential for neurotransmission, energy metabolism, nitrogen homeostasis, and redox balance. For example, b-alanine, a non-proteinogenic amino acid, functions as a neuromodulator and co-agonist of GABA and glycine receptors, thereby contributing to inhibitory signaling [39]. Glutamine plays a central role in the glutamate-glutamine cycle, replenishing pools of both excitatory and inhibitory neurotransmitters [40]. Glycine serves as a co-agonist at NMDA receptors and as an inhibitory neurotransmitter, and it also contributes to one-carbon metabolism and glutathione synthesis. Proline supports redox regulation and stress responses in mitochondria, whereas arginine is a precursor of nitric oxide and polyamines, which influence synaptic signaling and cell growth. The depletion of these amino acids likely reflects a metabolic shift away from anabolic support, antioxidant defense, and neurotransmitter buffering; such changes could exacerbate neuronal hyperexcitability and reduce stress resilience.

The *para*^*Shu*^-induced reorganization of the tryptophan metabolic network (Fig. 5) is of particular interest. It is possible that a shift away from serotonin synthesis reduces neuromodulation by this neurotransmitter. Given that reduced serotonin signaling has been associated with increased seizure susceptibility [41], the observed decrease in serotonin is likely to contribute to the seizure phenotypes observed in *para*^*Shu*^ mutants. Downregulation of the kynurenine pathway intermediate 3-hydroxykynurenine can be neuroprotective, as it limits oxidative stress and excitotoxicity by reducing conversion to the neurotoxic metabolite quinolinic acid [42, 43]. However, this shift may also impair NAD^+^ synthesis, which would have adverse effects on energy metabolism. In addition, elevations in the kynurenine pathway metabolite xanthurenate are associated with altered glutamatergic signaling and metabolic dysfunction, and this could promote neuronal hyperexcitability. In contrast, the indole pathway metabolite indolepropionate is a potent antioxidant, and its increase has been associated with neuroprotection and improved metabolic health. Collectively, these findings about differences in levels in metabolites in the tryptophan pathway suggest that it may be their relative balance that critically influences neuronal excitability and systemic homeostasis. Notably, a recent study of plasma samples from 18 pediatric patients with epilepsy and 11 age-matched healthy controls demonstrated that alterations in tryptophan metabolism are a hallmark of epilepsy [7]. This underscores the commonalities in metabolic changes in epileptic patients and hyperexcitable *Drosophila* VGSC mutants, and supports the notion that tryptophan metabolism will be a good target in developing therapies for sodium channelopathies and seizure disorders.

Collectively, our findings indicate that the *para*^*Shu*^ mutation induces a profound metabolic shift characterized by impaired carbohydrate metabolism, increased reliance on glycolysis, disrupted mitochondrial function, and altered regulation of amino acid metabolism and redox homeostasis. This metabolic reprogramming likely contributes to the severity of the hyperexcitable neural phenotype in *para*^*Shu*^ mutants, underscoring the bidirectional relationship between genetically driven neuronal hyperexcitability and metabolic abnormalities.

### 4.2. Implications for the roles of the immune response and inflammation in regulating *para*^*Shu*^ phenotypes.

Activation of the immune system and inflammation is increasingly recognized as a key determinant of seizure severity [44, 45]. During seizures, neuronal hyperactivity triggers the release of pro-inflammatory cytokines from neurons, glia, and immune cells. These mediators, in turn, can lower the seizure threshold by enhancing excitatory, and reducing inhibitory, neurotransmission. We previously found that reduced activity of glutathione S-transferase S1 (GstS1) mimics the seizure-suppressing effects of dietary ALA in *para*^*Shu*^ mutants [46]. GstS1 is a putative ortholog of mammalian hematopoietic prostaglandin D synthase (HPGDS) [47, 48], an enzyme involved in the biosynthesis of pro-inflammatory lipid mediators. Since both ALA and other ω−3 polyunsaturated fatty acids have anti-inflammatory effects in mammals, these findings suggest that GstS1 promotes inflammatory signaling that exacerbates seizure severity in *para*^*Shu*^ mutants, and that ALA antagonizes this process. This idea is supported by the observation that genes associated with inflammation and innate immune responses (including some that encode antimicrobial peptide genes) are strongly upregulated in *para*^*Shu*^ mutants and that this effect is reversed by loss of GstS1 function or supplementation of the diet with milk lipids [9, 23, 46].

A role for inflammation and immune function in modulating seizure severity in *para*^*Shu*^ mutants is further supported by several of the observed differences in metabolites. First, *para*^*Shu*^ mutants exhibit increased glycolytic activity. Glycolysis is tightly linked to proinflammatory immune responses because activated immune cells preferentially use glycolysis over oxidative phosphorylation to meet their energetic and biosynthetic demands. Second, succinate levels were reduced in *para*^*Shu*^ mutants, and this effect was reversed by ALA supplementation. In *Drosophila*, succinate is an immunometabolic signal and parallels its roles in mammalian immunity, i.e., stabilizing Sima/HIF pathways, promoting ROS-mediated microbial defenses, and maintaining redox homeostasis in hematopoietic progenitors [49, 50]. Third, N-methylnicotinamide (MeNAM) was among the most significantly decreased metabolites in *para*^*Shu*^ mutants and was restored by ALA supplementation. MeNAM actively regulates inflammation and innate immunity by suppressing NF-κB-dependent production of cytokines, enhancing prostacyclin signaling, reducing oxidative stress, and linking NAD^+^ metabolism to immune function. Fourth, cGMP levels were substantially higher in *para*^*Shu*^ mutants (2.4-fold vs. wild type) but lower following ALA supplementation (FC = 0.67, FDR = 0.051). In *Drosophila*, cGMP signaling activates NF-κB transcription factors such as Dif and Dorsal, which regulate both humoral and cellular immune responses [51]. Fifth, histamine levels were higher in *para*^*Shu*^ mutants but lower in these animals following ALA treatment. Although in *Drosophila* histamine is primarily a neurotransmitter involved in visual and mechanosensory transmission [52], in mammals it is known to play a critical role in inflammation and innate immunity [53]. Thus, our findings may reflect an evolutionarily conserved immunomodulatory role for histamine. Sixth, levels of pyridoxal, a vitamin B6 metabolite, was markedly decreased (FC = 0.514, FDR = 0.0287) in *para*^*Shu*^ mutants fed ALA. In vertebrates, vitamin B6 is essential for regulation of the innate immune system, functioning as a cofactor in enzymatic pathways that control cytokine production, macrophage activity, and lymphocyte proliferation. Dysregulation of B6 metabolism leads to aberrant inflammatory signaling, and through its role in the kynurenine pathway, vitamin B6 also influences the production of immunomodulatory metabolites [54]. Thus, ALA-induced changes in vitamin B6 metabolism might affect seizure severity in *para*^*Shu*^ mutants by modulating either neurotransmitter synthesis or innate immunity and inflammation.

### 4.3. Possible involvement of gut microbiota in the effects of *para*^*Shu*^ mutation and dietary ALA supplementation

We previously found that depletion of commensal gut bacteria markedly attenuates the seizure phenotype of *para*^*Shu*^ mutants [55], suggesting that bacterial metabolites or microbiota-driven host responses exacerbate behavioral hyperexcitability. The metabolomic analysis presented here strengthens this view by revealing significant alterations in metabolites of microbial origin. Notably, levels of SCFAs such as propionic acid and butyric acid were elevated 2.7-fold and 1.3-fold, respectively, in *para*^*Shu*^ mutants (Fig. 7A, B). These SCFAs are well-established products of bacterial fermentation of dietary products (carbohydrates and amino acids) [56], and they can enter systemic circulation to influence host physiology, including brain function [57]. Although SCFAs often exert beneficial effects, elevated propionate has been linked to pro-convulsant activity and altered neurotransmission in both animal models and humans [58, 59]. Our study also revealed increases in levels of microbial tryptophan metabolites, including tryptamine (1.7-fold) and indolepropionate (1.2-fold), which are known to modulate neuronal excitability and neuroinflammation [60, 61]. These findings suggest that the *para*^*Shu*^ mutation alters the gut environment, thereby modifying bacterial metabolism or populations and leading to changes in bioactive metabolites that influence seizure susceptibility.

Like the depletion of bacteria [55], dietary supplementation with ALA and genetic reduction of GstS1 activity suppressed seizures in *para*^*Shu*^ mutants [23, 46]. ALA supplementation reversed many *para*^*Shu*^-induced metabolic changes, including normalization of propionate and butyrate levels. Because these SCFAs are produced mainly by gut bacteria, this effect may be a consequence of a modulation of either the composition or metabolic activity of the microbiota, consistent with evidence that dietary ω-3 fatty acids alter the microbiota profile and SCFA production [62, 63]. The ability of bacterial depletion, ALA supplementation, and GstS1 suppression to reduce seizure severity suggests that these approaches may act on partially overlapping mechanisms to buffer the effects of microbiota-derived pro-seizure metabolites. ALA may counteract the effects of SCFA elevation by shifting microbial fermentation or host lipid signaling, whereas the suppression of the *Drosophila* ortholog of mammalian prostaglandin D synthase (GstS1) likely influences prostaglandin-dependent inflammatory pathways downstream of microbiota-host interactions.

Collectively, our results support a model in which the gut microbiota contribute to the severity of the *para*^*Shu*^ seizure phenotype by producing SCFAs, tryptophan derivatives, and other metabolites that influence neuronal excitability and inflammatory tone, and that interventions that modify the gut-host metabolic interface—through microbiota depletion, dietary fatty acid supplementation, or targeting prostaglandin synthesis—can attenuate seizure severity. Future studies using germ-free flies and targeted manipulation of the microbiota will be essential for identifying the specific bacterial taxa and metabolites mediating these effects.

## 5. Conclusion

This study demonstrates that a seizure-associated mutation in the *Drosophila* VGSC gene induces broad metabolic reprogramming encompassing energy metabolism, redox balance, amino acid and nucleotide pathways, and gut microbiota-derived metabolites. The partial normalization of these disturbances by dietary ALA highlights the potential of this ω-3 polyunsaturated fatty acid as a modulator of seizure-related metabolism. Furthermore, the involvement of immune and inflammatory pathways suggests that they represent an additional layer of regulation in seizure susceptibility. This metabolomic study carried out in *Drosophila* seizure mutants under strictly controlled genetic and environmental conditions provides a foundation for future studies to dissect contributions of metabolism, immunity, and microbiota to epilepsy pathophysiology, and for developing novel preventative and therapeutic strategies.

## Supplementary Material

Supplementary Files

This is a list of supplementary files associated with this preprint. Click to download.

• SupTable1Metabolite100325.docx

• SupTable2AllANOVA100425.docx

## Figures and Tables

**Table 1.** List of metabolites significantly affected by the *para^Shu^* mutation (A) and by dietary ALA supplementation (B).

A		
Shu-Ctrl / WT-Ctrl		
Metabolite	FC	FDR
Laurate	1.7032	1.8131e-05
**Propionic acid**	**2.652**	**4.4605e-05**
Inositol	0.75552	6.5209e-05
Xanthurenate*	1.4362	0.00026273
cGMP	2.4317	0.00041612
**N-methylnicotinamide**	**0.50196**	**0.00041612**
Arginine*	0.79643	0.00041612
Tryptamine*	1.7373	0.00088473
Nudifloramide	0.66979	0.00088473
Sedoheptulose 7-phosphate	0.69263	0.00088473
**Succinate**	**0.76745**	**0.00088473**
GMP	1.2756	0.00088473
Spermidine	0.83216	0.00088473
Pyruvate	1.4484	0.0010302
Glycine*	**0.75713**	**0.0010302**
CMP	1.5438	0.0013928
Nicotinamide riboside	0.52942	0.0017985
dAMP	1.6643	0.0024425
Fructose 6-phosphate	0.68543	0.0027042
Pantothenate	0.78695	0.0031457
alpha-Ketoglutarate	1.4932	0.003699
Nicotinic acid adenine dinucleotide	0.70015	0.003699
3-Hydroxyanthranilic acid*	0.78518	0.003699
Serotonin*	0.86251	0.003699
UMP	1.4767	0.0041324
Glucose 6-phosphate	0.66811	0.0051104
Pimelate	0.79427	0.0051652
Indolepropionate*	1.1947	0.0051652
Thymidine	1.758	0.0081491
dGDP	0.77616	0.008532
XMP	0.46705	0.012932
CDP	1.8856	0.012932
Lactate	1.3062	0.016151
beta-Alanine*	0.83125	0.016771
Histamine*	2.0081	0.016984
Butyric acid	1.2829	0.017975
Fumarate	0.48729	0.018805
Tryptophol*	0.81233	0.018805
**6-Phosphogluconate**	**0.82089**	**0.018805**
Gamma-aminobutyrate*	1.2513	0.018816
Proline*	**0.81779**	**0.019153**
3-Hydroxykynurenine*	0.76602	0.023844
O-Phosphoethanolamine	1.3542	0.024609
Homocysteine*	1.358	0.025338
Cadaverine*	0.85495	0.025865
Pentadecanoate	0.8513	0.026452
Xanthosine	0.61799	0.027825
**Stearate**	**0.77327**	**0.027825**
alpha-Ketoisovalerate*	1.2748	0.027877
Thymine	1.2917	0.029036
3-Hydroxypropionate	0.82353	0.030201
Heptanoic acid	1.3747	0.032302
dCMP	1.1582	0.043924
Glutamine*	0.88733	0.045465
Alanine*	0.9082	0.046378
B		
Shu-ALA / Shu-Ctrl		
Metabolite	FC	FDR
Linolenate	12.225	1.4808e-08
Linoleate	0.48097	0.0013089
**Stearate**	**1.2795**	**0.0019819**
**6-Phosphogluconate**	**1.3278**	**0.0042623**
**Propionic acid**	**0.65447**	**0.0078401**
Pyridoxal	0.51448	0.028726
**N-methylnicotinamide**	**1.5298**	**0.031009**
**Proline**	**1.2443**	**0.049725**
**Glycine**	**1.184**	**0.049725**
**Succinate**	**1.1469**	**0.049725**
Pterin	0.87857	0.049725

* Metabolites directly involved in amino acid metabolism. Metabolites shown in bold indicate those affected by both the *para^Shu^* mutation and ALA supplementation.

**Table 2:** Metabolic pathways significantly altered by the *para^Shu^* mutation.

	Total Compound	Hits	FDR	Impact
**Alanine, aspartate and glutamate metabolism**	23	10	0.00010605	0.80406
**Nicotinate and nicotinamide metabolism**	9	7	0.00059349	0.77357
**Pentose phosphate pathway**	22	9	8.9776e-05	0.72251
**Glycine, serine and threonine metabolism**	29	6	0.00078037	0.66696
**Arginine biosynthesis**	12	9	0.0032563	0.62857
**Tryptophan metabolism**	30	8	0.00045934	0.54863
**Pyrimidine metabolism**	40	15	0.0032563	0.52349
**Glutathione metabolism**	26	9	0.0066485	0.51293
**Arginine and proline metabolism**	29	6	0.00045934	0.51111
**Purine metabolism**	68	22	0.00010605	0.48849
**Citrate cycle (TCA cycle)**	20	10	0.00059349	0.43363
**Pyruvate metabolism**	23	7	0.0012616	0.46638
**Starch and sucrose metabolism**	14	4	0.0016183	0.41616
**Glycolysis / Gluconeogenesis**	26	8	0.0029461	0.40948
**Histidine metabolism**	9	2	0.007751	0.4
**Tyrosine metabolism**	33	5	0.00078037	0.38612
**Cysteine and methionine metabolism**	32	5	0.00073611	0.34704
**Propanoate metabolism**	20	7	8.9776e-05	0.33682

## Data Availability

The datasets generated and analyzed during the current study are available from the corresponding author upon reasonable request.

## Abbreviations

(ALA): α-linolenic acid
(CCM): central carbon metabolism
(GC-MS): gas chromatography-mass spectrometry
(MCFA): medium-chain fatty acid
(NAD^+^): nicotinamide adenine dinucleotide
(LC-MS): liquid chromatography-mass spectrometry
(LA): linoleic acid
(PPP): pentose phosphate pathway
(SMEI): severe myoclonic epilepsy of infancy
(SCFA): short-chain fatty acid
(TCA): tricarboxylic acid
(VGSC): voltage-gated sodium channel
